# Establishment of efficient Trichosanthes mottle mosaic virus-derived gene silencing in cucurbit plants

**DOI:** 10.1007/s44154-025-00238-5

**Published:** 2025-05-20

**Authors:** Cheng Chen, Zhu Fang, Min Du, Changkai Yang, Yukui Yang, Xueping Zhou, Xiuling Yang

**Affiliations:** 1 State Key Laboratory for Biology of Plant Diseases and Insect Pests, Institute of Plant Protection, Chinese Academy of Agricultural Sciences, Beijing, 100193 China; 2 Institute of Plant Protection, Sichuan Academy of Agricultural Science, Key Laboratory of Integrated Pest Management on Crops in Southwest, Ministry of Agriculture, Chengdu, 610066 China; 3 Yunnan Sinong Vegetables Seed Co., Ltd, Chuxiong, 651300 China; 4 State Key Laboratory of Rice Biology, Institute of Biotechnology, Zhejiang University, Hangzhou, 310058 China

**Keywords:** Trichosanthes mottle mosaic virus, Virus-induced gene silencing, Cucurbits, Flower

## Abstract

The Cucurbitaceae family includes a wide range of economically important fruits and vegetables; however, the laborious and highly inefficient genetic transformation efficacy of cucurbits has hindered the exploration of their gene functions. Virus-induced gene silencing (VIGS) technology, employed from the antiviral RNA silencing defense, has emerged as a viable alternative for high-throughput study of plant gene function. In this study, we successfully established a VIGS system utilizing Trichosanthes mottle mosaic virus (TrMMV), a new member of the genus *Tobamovirus*. We demonstrated the high efficacy and durability of gene silencing mediated by the TrMMV-VIGS vector in *Nicotiana benthamiana*, as well as in several cucurbit species, including *Cucurbita pepo*, *Cucumis sativus*, *C. lanatus*, and *C. melo*. The insertion of 90–400 bp fragments into the vector led to effective silencing of the target gene in both *C. sativus* and *C. melo*, with a notably higher silencing efficiency observed in *C. melo*. Furthermore, the TrMMV-VIGS vector induced a pronounced photobleaching phenotype in the flowers of *C. melo*, underscoring its potential application in functional genomic research concerning floral traits in this particular species. Taken together, the TrMMV-VIGS system developed herein will facilitate rapid and high-throughput identification of gene functions in cucurbit crops.

**Supplementary Information:**

The online version contains supplementary material available at 10.1007/s44154-025-00238-5.

## Introduction

The Cucurbitaceae family is one of the most genetically diverse plant families, encompassing a wide variety of economically important fruits and vegetables renowned for their nutritional value and delectable taste. Many Cucurbitaceae species also possess substantial pharmacological properties, such as antibacterial, antioxidant, and anti-cancer effects (Rolnik & Olas 2020; Yu et al. 2023). The rapid development of sequencing technologies and bioinformatics has accelerated the generation of genomes for over 20 cucurbit species, facilitating a deeper understanding of genome evolution and molecular breeding in cucurbit crops (Ma et al. 2022; Yang et al. 2023; Yu et al. 2023). While genome sequences have greatly aided gene identification in cucurbit, the labor-intensive and inefficient efficacy of genetic transformation in cucurbits has impeded the exploration of their gene functions (Chovelon et al. 2011; Feng et al. 2023; Geng et al. 2022; Pawelkowicz et al. 2024).

RNA silencing is a conserved antiviral defense that suppresses gene expression in a sequence-specific manner via small interfering RNA-mediated post-transcriptional gene silencing. Virus-induced gene silencing (VIGS) is an RNA silencing-based technology that has emerged as a viable alternative for high-throughput study of plant gene function without the need for genetic transformation. This approach involves infecting plants with a modified virus containing a partial fragment of the target gene, resulting in gene silencing and phenotypic defect due to the downregulation of the target gene (Yamamoto et al. 2021). Since the discovery of VIGS in 1995 (Kumagai et al. 1995), various viruses, including RNA viruses, DNA viruses, and satellites, have been developed as VIGS vectors to investigate the functions of target genes in either dicots or monocots (Ramegowda et al. 2014; Ratcliff et al. 2001; Ruiz et al. 1998). Currently, VIGS vectors have been employed to identify genes involved in plant growth, development, reproduction, and metabolism, and to study plant responses to biotic and abiotic stresses (Lange et al. 2013; Ramegowda et al. 2014; Burch-Smith et al. 2004).

The apple latent spherical virus (ALSV)-derived VIGS vector is the first reported VIGS vector to achieve silencing of the endogenous Phytoene desaturase (*PDS)* in *C. moschata*, *C. sativus*, *C. melo*, *Lagenaria siceraria*, *Citrullus lanatus* and *Luffa cylindrica *(Yamagishi and Yoshikawa 2022)*.* However, particle bombardment is required to deliver the target viral vector into plants (Igarashi et al. 2009; Yamagishi and Yoshikawa 2022). Subsequently, tobacco ringspot virus (TRSV) (Zhao et al. 2016) and tobacco rattle virus (TRV) (Bu et al. 2019) have been modified to induce gene silencing in cucurbits. Nevertheless, the manipulation and delivery methods of these two vectors and their limited silencing efficiency have restricted their application in functional genomic studies of cucurbits. Therefore, it is important to develop new VIGS vectors that consistently achieve effective silencing in cucurbits.

Tobamoviruses are a group of single-stranded RNA viruses that include several cucurbit-infecting type species, such as cucumber green mottle mosaic virus (CGMMV) and cucumber fruit mottle mosaic virus (CFMMV). Previously, we identified a distinct tobamovirus, Trichosanthes mottle mosaic virus (TrMMV), from symptomatic *Trichosanthes kirilowii* plants, a member of the Cucurbitaceae family (Chen et al. 2022). We successfully constructed a full-length infectious clone of TrMMV and demonstrated its systemic infection in *Nicotiana benthamiana*, as well as in various cucurbit plants, including *T. kirilowii*, *Cucumis sativus*, *C. melo*, *Luffa aegyptiaca*, *C. lanatus*, and *Cucurbita pepo* (Chen et al. 2022). The broad host range of TrMMV makes it a promising candidate for developing VIGS vector in cucurbit plants. In this study, TrMMV has been modified as a VIGS vector, and an efficient VIGS system was successfully established in diverse cucurbit plants using the TrMMV-derived VIGS vector.

## Results

### Modification of TrMMV into a gene-silencing vector

**Fig. 1:**
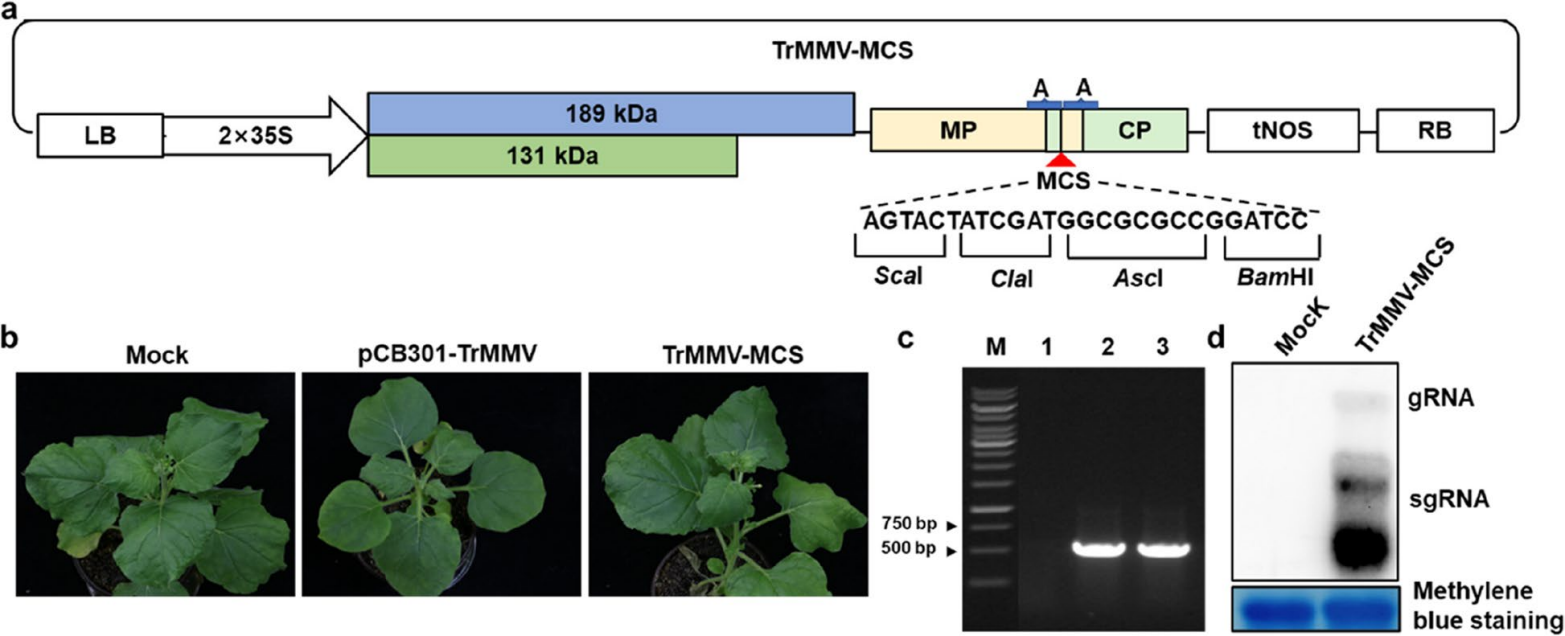
Construction of a Trichosanthes mottle mosaic virus (TrMMV)-derived vector. **a** Schematic diagram of the TrMMV-MCS vector. The modified TrMMV-MCS vector was constructed based on the infectious clone of pCB301-TrMMV. Restriction sites that can be used to clone the fragment of interest are shown as indicated. The 131-kDa and 189-kDa replicase proteins, along with the movement protein (MP) and coat protein (CP) encoded by TrMMV, are represented. Region A corresponds to the TrMMV subgenomic CP promoter, encompassing a 211-bp sequence from the end of 5'MP to the start of 3'CP. 35S, the cauliflower mosaic virus promoter; tNos, Nos terminator. LB and RB indicate left border and right border of T-DNA, respectively. **b** Infectivity of the modified TrMMV-MCS vector in *Nicotiana benthamiana*. Symptoms were captured at 16 days post infiltration (dpi). **c** RT-PCR detection of the systemic infection of TrMMV-MCS. RNA extracted from the systemic leaves of the represented *N. benthamiana* was used for cDNA synthesis and PCR detection. 1, *N. benthamiana* plants inoculated with the pCB301 empty vector; 2, *N. benthamiana* plants inoculated with pCB301-TrMMV; 3, *N. benthamiana* plants inoculated with TrMMV-MCS. **d** Northern blot analysis of TrMMV genomic RNA. 30 μg of total RNA extracted from *N. benthamiana* plants infiltrated with pCB301 (Mock) or TrMMV-MCS were analyzed. Viral genomic RNA was detected using a DIG-labeled TrMMV CP-specific probe. Methylene blue staining was used to visualize total RNA loading. gRNA, genomic RNA; sgRNA, subgenomic RNA

To modify TrMMV into a gene-silencing vector, we introduced multiple cloning sites (MCS) between the movement protein (MP) and coat protein (CP) of TrMMV and denoted the resulting vector as TrMMV-MCS (Fig. 1A). The modified vector was transformed into *Agrobacterium tumefaciens* EHA105 and agroinfiltrated into the leaves of *N. benthamiana*. *N. benthamiana* plants infiltrated with the pCB301 vector or the infectious clone of TrMMV (pCB301-TrMMV) were used as controls. Compared to those infiltrated with the infectious clone of TrMMV, the modified vector caused a milder symptom in the systemic leaves of inoculated *N. benthamiana* plants at 16 dpi (Fig. 1B). Reverse transcription PCR (RT-PCR) and Northern blot analysis demonstrated the infection of TrMMV-MCS in *N. benthamiana* plants (Fig. 1C and D).

### The TrMMV-MCS vector induces gene silencing in *N. benthamiana*

**Fig. 2:**
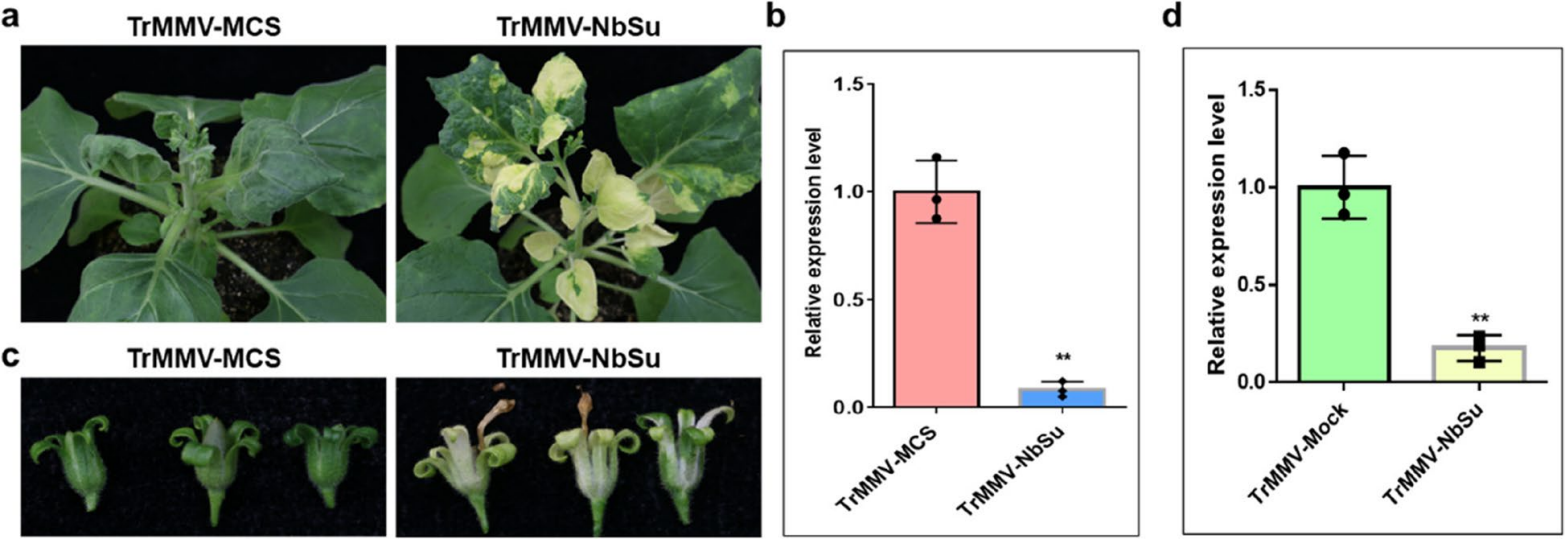
Establishment of TrMMV-induced gene silencing in *N. benthamiana* plants. **a** Silencing phenotype of *N. benthamiana* mechanically inoculated with the sap obtained from *N. benthamiana* plants agroinoculated with TrMMV-NbSu. Photos were captured at 18 dpi. **b** RT-qPCR analysis of the silencing efficiency of the endogenous *Su* gene in *N. benthamiana.* **c** Silencing phenotype of the calyx in *N. benthamiana* inoculated with TrMMV-NbSu or TrMMV-MCS. Photos were captured at 50 dpi. **d** RT-qPCR detection of the silencing efficiency of the endogenous *Su* gene in the calyx of *N. benthamiana*. Relative expression levels of the *NbSu* gene in (**a**) and (**c**) were determined using *Su*-specific primers and normalized to the *Actin* gene. The relative expression level of *NbSu* in TrMMV-MCS samples were set to 1. Significant differences were determined using Student’s *t*-test. ** indicates *p* < 0.01. Data represents mean value of triplicates from three biological repeats. Error bars represent standard deviation

To investigate whether the TrMMV-MCS vector can induce effective gene silencing, we constructed a TrMMV-NbSu vector by incorporating the *NbSu* fragment of *N. benthamiana* into the TrMMV-MCS vector via one-step assembly. The *Su* gene encodes a component of the magnesium chelatase complex, which is essential for the biosynthesis of chlorophyll II (Kjemtrup et al. 1998). If TrMMV-NbSu effectively suppress *NbSu* expression, it would result in a white leaf phenotype due to the inhibition of chlorophyll II biosynthesis. *A. tumefaciens*-mediated inoculation showed that TrMMV-NbSu vector could induce silencing of *NbSu* in the upper non-infiltrated systemic leaves of *N. benthamiana* at 16 dpi, although spotted silencing was observed in most leaves (Fig. S1). Control plants infiltrated with TrMMV-MCS remained green (Fig. S1). When the leaves exhibiting silencing phenotypes were rubbed onto healthy *N. benthamiana* plants, the systemic leaves of the inoculated *N. benthamiana* displayed robust silencing phenotypes at 10-–11 dpi, characterized by significant chlorosis and yellowing in the majority of leaves (Fig. 2A). Reverse transcription quantitative real-time PCR (RT-qPCR) analysis revealed a 92% reduction in the expression of endogenous *Su* mRNA in *N. benthamiana* (Fig. 2B). Notably, the silenced *N. benthamiana* plants also displayed gene silencing in the calyx, as evidenced by the bleached calyx (Fig. 2C). RT-qPCR analysis confirmed the persistent reduction of *Su* mRNA level in the calyx of the inoculated *N. benthamiana* plants (Fig. 2D), highlighting the potential of the TrMMV-NbSu vector to achieve long-lasting silencing effects.

### The TrMMV-MCS vector induces gene silencing in multiple cucurbit plants

**Fig. 3:**
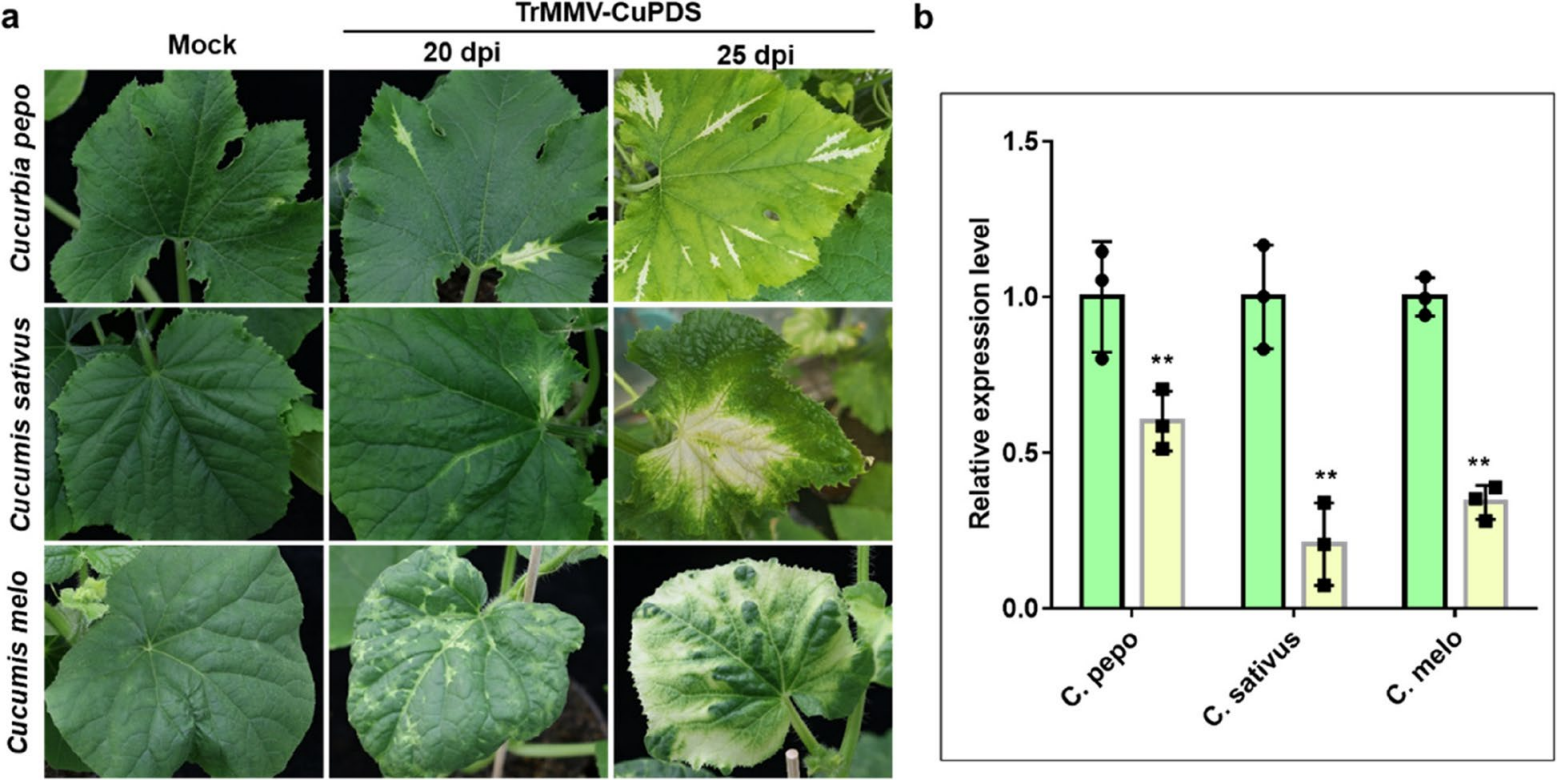
Establishment of TrMMV-induced gene silencing in cucurbit plants. **a** Silencing phenotype of CuPDS observed in* C. pepo*, *C. sativus,* and *C. melo*. Photos were captured at 20 dpi and 25 dpi, respectively. **b** RT-qPCR results showing the relative expression levels of the endogenous *PDS* genes in cucurbit plants at 25 dpi. Relative expression levels of *PDS* were assessed using *PDS*-specific primers and normalized to Actin. The relative expression level of *CuPDS *in TrMMV-MCS samples were set to 1. Significant differences were determined using Student’s *t*-test. ** indicates *p* < 0.01. Data represents mean value of triplicates from three biological repeats. Error bars represent standard deviation. At least 10 plants were used for each treatment

To assess the feasibility of the TrMMV-MCS vector on cucurbit plants, we compared the sequences of the *PDS* gene of different cucurbit plant species, selected, cloned, and inserted a conserved 213-bp PDS fragment into the TrMMV-MCS vector to generate the TrMMV-CuPDS vector. *Agrobacterium* harboring the TrMMV-CuPDS vector was infiltrated into *Cucurbia pepo*, *Cucumis sativus*, and *C. melo* plants to test its ability to induce *PDS* silencing in these cucurbit hosts. The results showed that TrMMV-CuPDS induced a characteristic gene silencing phenotype of leaf photobleaching in all inoculated *C. pepo, C. sativus* and *C. melo* plants at approximately 20 dpi, and in *Citrullus lanatus* at 30 dpi (Fig. 3A and Fig. S2). RT-qPCR analysis confirmed a significant suppression of the endogenous *PDS* gene in the tested cucurbit plants (Fig. 3B).

### Effect of insert size on TrMMV-MCS-mediated gene silencing

**Fig. 4:**
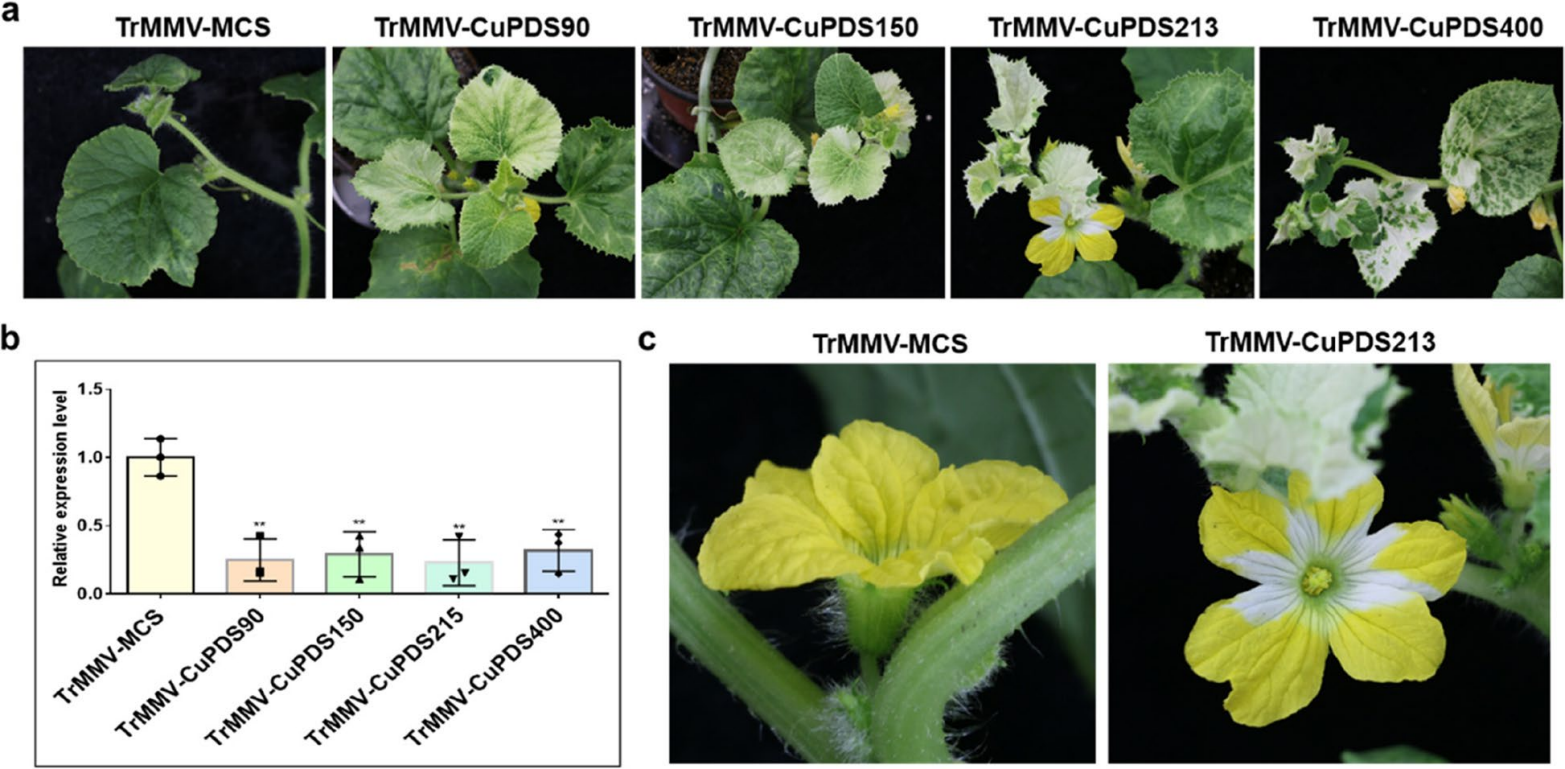
Effect of the length of the insert on silencing of the *PDS* gene mediated by the TrMMV-MCS vector. **a** Phenotype of *C. melo* inoculated with TrMMV-CuPDS vectors carrying PDS fragments of different sizes. **b** RT-qPCR analysis of the expression of the endogenous *PDS* gene in *C. melo*. **c** Photobleaching phenotype of *C. melo* flower resulting from the TrMMV-CuPDS213-induced silencing of *PDS*. ** indicates *p* < 0.01. Data represents mean value of triplicates from three biological repeats. Error bars represent standard deviation. At least 12 plants were used for each treatment

To investigate the impact of different insert fragment sizes on the TrMMV-VIGS vector, we constructed additional TrMMV-CuPDS vectors with insertions of 90 bp, 150 bp, and 400 bp of the CuPDS fragment into the MCS, respectively. *A. tumefaciens*-mediated inoculation revealed efficient infection of *C. sativa* by all vectors. However, the *PDS* silencing phenotype, characterized by leaf photobleaching, was observed only in a few leaves of *C. sativa*, with the most pronounced silencing phenotype observed in *C. sativa* plants inoculated with TrMMV-CuPDS150 (Fig. S3). In the case of *C. melo*, efficient infection was achieved with vectors containing different fragments of TrMMV-CuPDS, and most systemic leaves displayed complete photobleaching (Fig. 4A). RT-qPCR analysis demonstrated efficient silencing of the endogenous *PDS* gene in *C. melo*, with silencing efficiencies ranging from 56.5% to 89.5% and an average silencing efficiency of 72.9% (Fig. 4B). Notably, the TrMMV-CuPDS213 vector exhibited superior silencing effects, with obvious photobleaching observed in the flowers of the silenced *C. melo* plants (Fig. 4C). These results indicate that efficient silencing of endogenous genes could be achieved by inserting 150 or 213-bp fragments and the TrMMV-VIGS vector is more suitable for conducting gene silencing research in *C. melo* compared to other tested cucurbit crops.

## Discussion

Cucurbits contain many economically important vegetable crops and medicinal plants. Although the genomes of many species have been sequenced, the function of most of their genes remains unknown. The long-term genetic transformation process and low transformation efficacy impede the functional analysis of cucurbit genes, thereby limiting the breeding of high-yield and high-quality varieties (Fang et al. 2021). VIGS is widely used to study the function of plant genes due to its unique advantages. Development of an efficient VIGS vector is essential to accelerate research on gene function in cucurbit crops.

Key factors to consider in developing a VIGS vector include stable, high silencing efficacy of target genes, long-lasting silencing phenotype, and ease of manipulation. In this study, the TrMMV-VIGS vector was constructed by taking advantage of the subgenomic promoter of the viral CP to ensure efective infection of the modified TrMMV vector. Efficient silencing of target genes was observed in *N. benthamiana* and all the four tested cucurbit plants, though the silencing phenotype varied among different plant species. Interestingly, the silencing efficiency of the modified TrMMV vector in *N. benthamiana* was significantly improved by mechanical inoculation compared to agroinfiltration, and the silencing phenotype persisted in the calyx. The long-lasting silencing phenotype induced by the TrMMV-derived VIGS was also demonstrated in *C. melo*, where the flowers exhibited notable photobleaching, suggesting that TrMMV-derived VIGS is stable over time.

Theoretically, a DNA fragment with a minimum of 23 nucleotides, bearing 100% identity to a targeted gene, can initiate silencing. However, in most cases, longer identical sequences are used, and the upper limit for insert fragment size varies depending on different viruses (Burch-Smith et al. 2004). Analysis of the effects of different insertion sizes of target fragments into the TrMMV-VIGS vector showed that all the tested insertions could induce silencing of the endogenous *PDS*, with better photobleaching phenotypes observed for insertion sizes of 150 bp or 213 bp. A delayed silencing phenotype was observed with an insertion size of 400 bp, likely due to the longer insertion fragment restricting the intercellular movement of the virus.

Previously, several VIGS vectors derived from ALSV, TRSV, TRV, CGMMV, and CFMMV have been reported for use in cucurbit crops; however, they have not been widely applied in functional analyses of cucurbit genes (Bu et al. 2019; Igarashi et al. 2009; Liu et al. 2020; Zhao et al. 2016). Compared to those developed VIGS vectors, the TrMMV-derived VIGS system offers several distinct advantages. Firstly, unlike ALSV, TRSV, and TRV, which have bipartite genomes requiring the mixing of different components prior to inoculation, TrMMV possess a monopartite genome, simplifying the manipulation process. Moreover, ALSV proteins are expressed through proteolytic processing of its polyprotein, necessitating an in-frame fusion to insert target sequences of interest into the ALSV vector. In contrast, the monopartite genome of TrMMV enables a more straightforward approach to vector construction. Secondly, TrMMV can infect a broader range of cucurbit hosts than TRSV and TRV. The TrMMV-VIGS vector has been demonstrated to effectively infect and induce target gene silencing in various cucurbit species, including *C. pepo, C. sativus*, *Citrullus lanatus,* and *C. melo.* In contrast, TRSV is unable to infect *C. pepo* or *C. lanatus*, and TRV-induced gene silencing in cucurbits has only been reported in cucumber and oriental melon. CGMMV was the first tobamovirus developed to induce gene silencing in cucurbits including *C. lanatus, C. melo, C. sativus,* and *Lagenaria siceraria,* but robust silencing phenotype was only observed in the leaves of bottle gourd, and no data are available regarding silencing efficacy in the flowers of the tested cucurbits. Collectively, the TrMMV-VIGS vector developed in this study exhibited unique advantages, including ease manipulation, high and long-lasting silencing efficacy, a broad host range, and the ability to induce *PDS* silencing in flowers. Further exploring its potential utility in understanding the genes controlling various floral traits would yield valuable insights into development of cucurbits.

## Conclusion

In summary, we have successfully developed a VIGS system based on a new member of the genus *Tobamovirus*. We demonstrated efficient and long-lasting gene silencing effects of the TrMMV-VIGS vector in *N. benthamiana* and several cucurbit plants, including *C. pepo, C. sativus, C. lanatus,* and *C melo*. The insertion of 90–400 bp fragments into the vector resulted in effective silencing of the target gene in both *C. sativus* and *C. melo*, with higher silencing efficiency observed in *C. melo*. Furthermore, the TrMMV-VIGS vector induced a noticeable photobleaching phenotype in the flowers of *C. melo*, highlighting its promising application in functional genomic research related to floral traits in this species.

## Materials and methods

### Plant growth

*N. benthamiana*, *C. pepo, C. sativus, C. lanatus,* and *C. melo* were sown and the sprouted seedlings were transferred to separate pots. Plants were grown at 25 ℃ in an insect-free greenhouse under a 16-h/8-h photoperiod.

### Construction of the TrMMV-MCS vector

To obtain the clone pCB301-TrMMV-CP (TCG) containing a mutation from"ATG"to"ACG"mutation in the start codon of the TrMMV *CP* gene, the pCB301-TrMMV plasmid was used as the template for reverse PCR amplification with primers CP-TCG-F/CP-TCG-R (Table S1). The resulting fragment was digested with *DpnI* and purified for homologous recombination ligation. The desired mutation in the resulting clone was confirmed by sequencing.

To generate the TrMMV-MCS vector, three primer sets, TrMMV-VIGS-insert1 F/TrMMV-VIGS-insert 1R, TrMMV-VIGS-insert2 F/TrMMV-VIGS-insert 2R, and TrMMV-VIGS-insert3 F/TrMMV-VIGS-insert3R, were designed to amplify three fragments designated as insert1, insert2 and insert3. For insert1 and insert2, pCB301-TrMMV-CP (TCG) was used as the template to obtain the N-terminal 5953-bp fragment of the TrMMV genome, containing 78 nt of CP with the start codon modified from ATG to ACG. The sequence AGTACTATCGAT, encompassing two restriction sites (*Sca*I and *Cla*I), was introduced to the 5' end of insert2. Insert3, covering the 5743–6524 nt sequence of TrMMV, was amplified using the pCB301-TrMMV plasmid as template, encompassing the predicted promoter of CP, CP, and 3'UTR. The sequence GGCGCGCCGGATCC, containing two restriction sites (*Asc*I and *BamH*I), was attached to the 3' end of insert3. After purification, the insert1, insert2, and insert3 fragments were ligated into the pCB301 plasmid linearized by *Stu*I and *Sma*I using homologous recombination. The recombinant TrMMV-MCS clone was screened and confirmed by DNA sequencing.

### Construction of TrMMV-VIGS-derived vector

Primers NbSu215-insertF/NbSu215-insertR (Table S1) were used to amplify the 215-bp fragment of *NbSu* from the cDNA of *N. benthamiana*. The conserved PDS regions of *C. sativus, C. melo*, and *C. pepo* were selected, and the PDS fragments of 90 bp, 150 bp, 213 bp and 400 bp were amplified from the cDNA of *C. sativus* using primers CuPDS90-insertF/CuPDS90-insertR, CuPDS150-insertF/CuPDS150-insertR, CuPDS213-insertF/CuPDS213-insertR and CuPDS400-insertF/CuPDS400-insertR, respectively. The TrMMV-MCS plasmid was linearized with *Sca*I and *BamH*I and ligated with purified NbSu215, CuPDS90, CuPDS150, CuPDS213 and CuPDS400 via homologous recombination. The resulting plasmids, TrMMV-NbSu, TrMMV-CuPDS90, TrMMV-CuPDS150, TrMMV-CuPDS213, and TrMMV-CuPDS400 were verified by sequencing and transformed to *A. tumefaciens* strain EHA105 by electroporation. The accession numbers of *NbSu* and *CuPDS* are XM_019632045 and KX650422, respectively.

### Agroinoculation

*A. tumefaciens* containing each construct was cultured overnight at 28℃. The culture was collected and resuspended with infiltration buffer (10 mM MgCl_2_, 10 mM MES (pH 5.6), and 200 μM acetosyringone) to an optimal density of 1.0 (OD_600_ = 1.0). Agroinoculation of *N. benthamiana*, *C. pepo, C. sativus, Luffa cylindrica, C. lanatus*, and *C. melo* plants was conducted as previously described (Chen et al. 2022). For mechanical inoculation, sap extracted from the *N. benthamiana* leaves showing photobleaching phenotype was used to mechanically inoculate healthy *N. benthamiana* leaves as described (Ma et al., 2021).

### RNA extraction, RT-PCR and qRT-PCR

Total RNA was isolated from 50 to 100 mg of frozen plant leaf tissues using the FastPure Plant Total RNA Isolation Kit, Vazyme, China) according to the manufacturer’s instructions. First-strand cDNA was synthesized by reverse transcription of 1 μg of RNA with the oligo(dT) primer using the TaKaRa RNA PCR kit (AMV) Ver.3.0 (TaKaRa, Japan). Target fragment amplification was performed with TransStart® FastPfu DNA polymerase (Transgen Biotech, Beijing). Primers TrMMV-F and TrMMV-R, expected to amplify an amplicon of approximately 500 bp, were used to detect TrMMV infection. To detect the silencing efficacy of target genes, RT-qPCR was conducted using the LightCycler®96 Instrument (Roche, Germany). The *actin* gene was used an internal control. The relative expression of target genes was quantified using the 2^−ΔΔCT^ method, and the data presented are means of triplicates from three independent biological repeats in one representative experiment (Sun et al. 2023).

### Northern blot

For detection of genomic RNA of TrMMV-VIGS vector, 30 μg of total RNA extracted from mock plants or upper noninfiltrated leaves of *N. benthamiana* infiltrated with TrMMV-MCS was separated on a 1.5% formaldehyde-agarose gel and transferred to Hybond N ^+^ membranes (Cytiva, Marlborough, Massachusetts, USA). The membrane was hybridized with a digoxigenin-labeled probe targeting the CP of TrMMV, which was made using the PCR DIG probe synthesis kit (Roche Diagnostic, Basel, Switzerland). The resulting signal was detected using a using a DIG High Prime DNA Labeling and Detection Starter Kit according to the manufacturer’s instructions (Roche) and visualized with a chemiluminescence detection system (Tianneng, Shanghai, China).

## Supplementary Information

Additional file 1: Fig. S1. Gene silencing phenotype of *N. benthamiana* triggered by TrMMV-NbSu. Fig. S2. Silencing phenotype of *CuPDS* observed in *Citrullus lanatus*. Fig. S3. Silencing phenotype of *C. sativus* inoculated with TrMMV-CuPDS vectors carrying PDS fragments of different lengths.

## Data Availability

Not applicable.

## Abbreviations

VIGS: Virus-induced gene silencing
TrMMV: Trichosanthes mottle mosaic virus
Su: Sulfur allele
PDS: Phytoene desaturase
PVX: Potato virus X
TRV: Tobacco rattle virus
TRSV: Tobacco ringspot virus
CGMMV: Cucumber green mottle mosaic virus
CFMMV: Cucumber fruit mottle mosaic virus
ALSV: Apple latent spherical virus
MCS: Multiple cloning sites
CP: Coat protein
MP: Movement protein
RT-PCR: Reverse transcription-polymerase chain reaction
RT-qPCR: Reverse transcription quantitative real-time PCR
mRNA: Messenger RNA
bp: Base pair
