# Supplementary material for: Establishment of efficient Trichosanthes mottle mosaic virus-derived gene silencing in cucurbit plants
Source: Stress Biol. 2025 May 20;5(1):35. doi: 10.1007/s44154-025-00238-5 (PMC12092892; doi:10.1007/s44154-025-00238-5)
Supplement: Supplementary file 1 — Additional file 1: Fig. S1. Gene silencing phenotype of N. benthamiana triggered by TrMMV-NbSu. Fig. S2. Silencing phenotype of CuPDS observed in Citrullus lanatus. Fig. S3. Silencing phenotype of C. sativus inoculated with TrMMV-CuPDS vectors carrying PDS fragments of different lengths. [file 44154_2025_238_MOESM1_ESM.docx]

**Table S1** Primers used for construction of TrMMV-VIGS vectors and RT-qPCR

| Primer names | Primer sequences（5'-3'） |
| --- | --- |
| CP-TCG-F | GCGGGCACCAAAGCCCAATTACAGCAAATGTCGTTCCAC |
| CP-TCG-R | GCTGTAATTGGGCTTTGGTGCCCGCCACAGAG |
| TrMMV-VIGS-insert1F | GTTCATTTCATTTGGAGAGGATGGGGATAAAAGTATTTAAGCACA |
| TrMMV-VIGS-insert1R | CATCAACCCTTCGTCGACCCAAAGGGTCT |
| TrMMV-VIGS-insert2F | GGGTCGACGAAGGGTTGATGGTTCATAC |
| TrMMV-VIGS-insert2R | CCGGCGCGCCATCGATAGTACTATTATAAAGATCTAAATAAGGGAAAAAGG |
| TrMMV-VIGS-insert3F | TACTATCGATGGCGCGCCGGATCCTCTTATGTTTCTAAGTCTGGTTTGAACC |
| TrMMV-VIGS-insert3R | TGGAGATGCCATGCCGACCCTGGGGTCCTTACCCAGGACA |
| QP-TrMMV-F | AGTCGCTCGTTGGCTTGTTA |
| QP-TrMMV-R | GACAGCATTCAGCGCTTCAC |
| NbSu215-insertF | GATCTTTATAATAGTCAGGGCAGAGTCAAGGGAG |
| NbSu215-insertR | AAACATAAGAGGATCATTCAGCAAAAGACATAACTTCATC |
| CuPDS90-insertF | GATCTTTATAATAGTATGCTTACTTGGCCAGAGA |
| CuPDS90-insertR | AAACATAAGAGGATCATTATCTTGAGCCTCAACATAGGA |
| CuPDS150-insertF | GATCTTTATAATAGTGATATGGGCTATTTTAAGGAACAAC |
| CuPDS150-insertR | AAACATAAGAGGATCCCCCGACTTCTCATCCACTC |
| CuPDS213-insertF | GATCTTTATAATAGTATGCTTACTTGGCCAGAGAAAA |
| CuPDS213-insertR | AAACATAAGAGGATCAATGCATTGCATAGAAAGTTCATCG |
| CuPDS400-insertF | GATCTTTATAATAGTTTTGGGGCTTATCCCAATGTGC |
| CuPDS400-insertR | AAACATAAGAGGATCAAATGCATTGCATAGAAAGTTCATC |
| QP-NbActin-F | AAAGACCAGCTCATCCGTGGAGAA |
| QP-NbActin-R | TGTGGTTTCATGAATGCCAGCAGC |
| QP-NbSu-F | TCTCGCTCCTCTAAATCTTCCA |
| QP-NbSu-R | CTTGAGCAGGGCTGACTTCC |
| QP-CuActin-F | ATGGTCAAGGCTGGATTTGC |
| QP-CuActin-R | TGAGCTTCATCACCAACATAGGC |
| QP-CuPDS-F | TGTGTGGATTACCCTAGACC |
| QP-CuPDS-R | CCAAGCTGCTACCTTTCCAC |


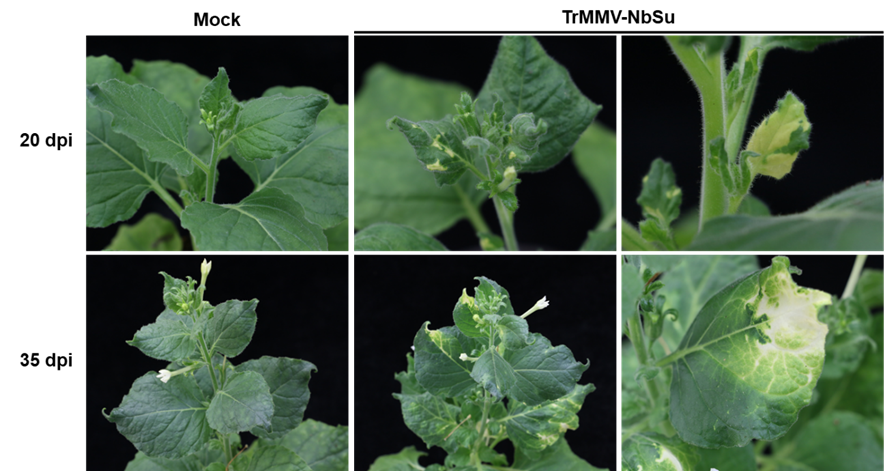


**Fig. S1** Gene silencing phenotype of *N. benthamiana* triggered by TrMMV-NbSu. *N. benthamiana* plants were agroinoculated with *Agrobacterium tumefaciens* cells harboring TrMMV-NbSu. Plants agroinoculated with TrMMV-MCS were used as mock controls.


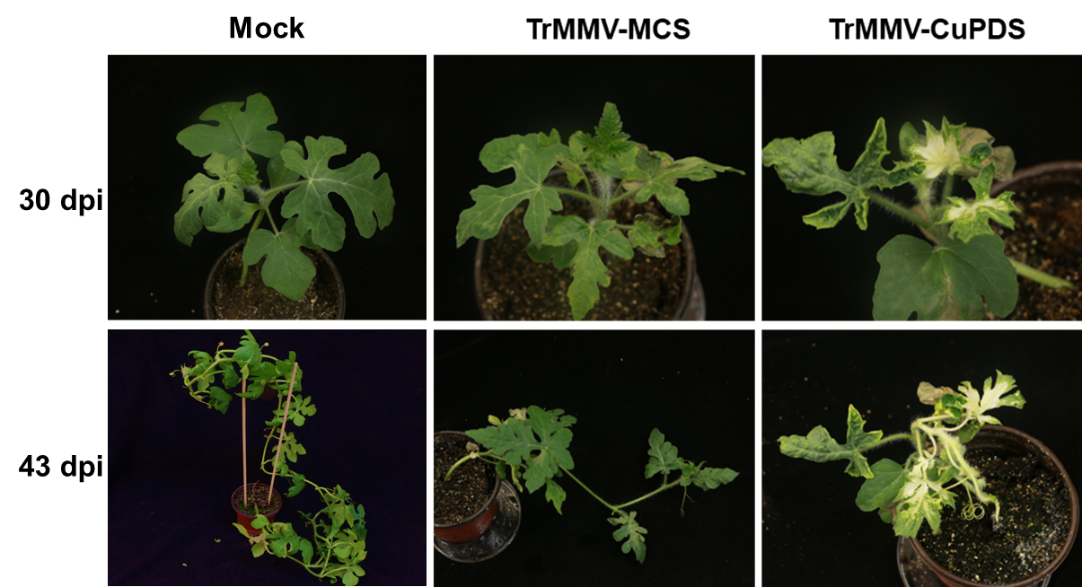


**Fig. S2** Silencing phenotype of CuPDS observed in *Citrullus lanatus*.


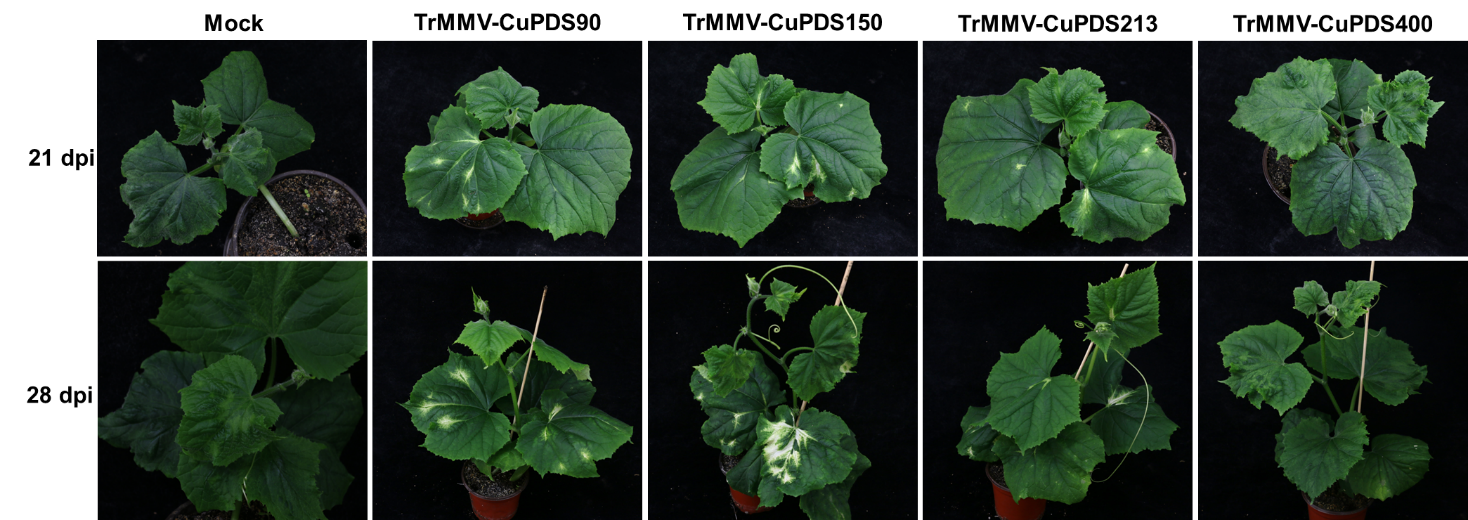


**Fig. S3** Silencing phenotype of *C. sativus* inoculated with TrMMV-CuPDS vectors carrying PDS fragments of different lengths.
